# The Effect of Dexamethasone Treatment on COVID-19 Prognosis in Cancer Patients

**DOI:** 10.3390/vaccines10111798

**Published:** 2022-10-26

**Authors:** Lina Souan, Zienab Al-Khairy, Maysaa’ Adnan Al-Binni, Abdelkader Battah, Maher A. Sughayer

**Affiliations:** 1 Department of Pathology & Laboratory Medicine, King Hussein Cancer Center, Amman 11941, Jordan; 2 Department of Clinical Laboratory Science, The University of Jordan, Amman 11942, Jordan; 3 Department of Pathology, Microbiology and Forensic Medicine, School of Medicine, The University of Jordan, Amman 11942, Jordan

**Keywords:** COVID-19, cancer patients, dexamethasone, corticosteroid, therapy, SARS-CoV-2

## Abstract

Background: Dexamethasone is used to treat cancer, relieve chemotherapy-induced nausea and vomiting, enhance cancer patients’ appetites, and treat COVID-19 patients. There is little evidence of the impact of a dexamethasone treatment plan on the severity of COVID-19 infections in cancer patients. This study explores whether dexamethasone treatment plan influences the severity of COVID-19 in dexamethasone-treated cancer patients. Methods: The medical records of 108 cancer patients receiving dexamethasone at King Hussein Cancer Center with a COVID-19 infection and 343 without corticosteroid treatment were reviewed. Patients on dexamethasone within seven days before infection, after infection, or both were included. Ventilation support, hospitalization, and mortality within 28 days of a COVID-19 diagnosis were key severity factors. Results: We found that dexamethasone before a COVID-19 infection increased the risk of requiring ventilation assistance and mortality within 28 days by a factor of 5.8 (2.8–12.0) relative to control (*p* < 0.005). Continuing dexamethasone treatment after a COVID-19 infection, or starting it after infection, had a risk factor equivalent to control. Conclusion: Our data showed that dexamethasone therapy protocol affected COVID-19 prognoses in cancer patients, and it is preferable to not discontinue therapy after infection. A rigorous prospective comparison between early and late dexamethasone dosing is needed to determine the best protocol for treatment.

## 1. Introduction

The novel virus that emerged in China at the end of 2019, coronavirus disease 2019 (COVID-19), was identified in patients with severe pneumonia and flu-like symptoms, and was classified as a novel strain of severe acute respiratory syndrome coronavirus 2 (SARS-CoV-2) [1,2,3,4]. The symptoms of COVID-19 vary from individual to individual, from asymptomatic to acute respiratory distress syndrome and a characteristic hyper-inflammatory response that could lead to multiple organ failures [5,6,7,8,9].

The COVID-19 disease progresses through four stages, beginning with an infection in the upper respiratory tract and followed by dyspnea and pneumonia and progressing to a cytokine storm and subsequent hyper-inflammatory state that causes more deleterious effects. It ends with either the recovery of the patient or death that is linked to advanced age, the presence of comorbidities, the severity of the disease, and the progression of respiratory failure [6,9,10]. It was postulated that limiting or preventing the cytokine storm may decrease inflammation-induced damage to the respiratory tract and hence reduce progression to respiratory failure and death in patients with COVID-19 [11,12]. Dexamethasone is a synthetic glucocorticoid drug that is routinely prescribed to cancer patients as a direct chemotherapeutic agent in certain hematological cancers, such as leukemia or multiple myeloma [13,14]. It is also used as an effective salvage therapy option for relapsed/refractory acute lymphoblastic leukemia (ALL) and as a supportive care co-medication to reduce the unfavorable effects of anticancer agents for cancer patients undergoing standard-care pemetrexed/platinum doublet chemotherapy [15]. Dexamethasone was found to decrease edema associated with tumors, and some studies suggested that it has the potential to significantly inhibit tumor growth up to 26 days after initiation of treatment. This is due to the immunosuppressive and anti-inflammatory effects of dexamethasone [16]. A recent controlled, open-label, randomized trial evaluating dexamethasone therapy in patients hospitalized with COVID-19 (RECOVERY) found that patients infected with SARS-CoV-2 and were treated with dexamethasone resulted in a lower 28-day mortality among those who were receiving either invasive mechanical ventilation or oxygen alone [11]. Active cancer can enhance susceptibility to the COVID-19 infection in immunocompromised states because cancer patients already have significantly weakened and altered immune systems due to cancer therapy, primary illness etiology, and disease extent. This would put them in a state where they are more likely to contract severe illnesses and die when exposed to the virus [5,17]. Death rates and critical symptoms were the worst among 105 COVID-19 cancer patients studied in Hubei Province, China, according to a recent study [18]. Meanwhile, another study by Memorial Sloan Kettering Cancer Center demonstrated that recent cancer treatment was not associated with adverse COVID-19 outcomes [19]. As of now, there is no adequate research on the effects of dexamethasone on cancer patients infected with the SARS-CoV-2 virus regarding the treatment or management of COVID-19. Hence, this study aims to determine whether dexamethasone treatment before or after a COVID-19 diagnosis, or both, influences COVID-19 severity indices in dexamethasone-treated cancer patients.

## 2. Materials and Methods

### 2.1. Study Design

**Figure 1:**
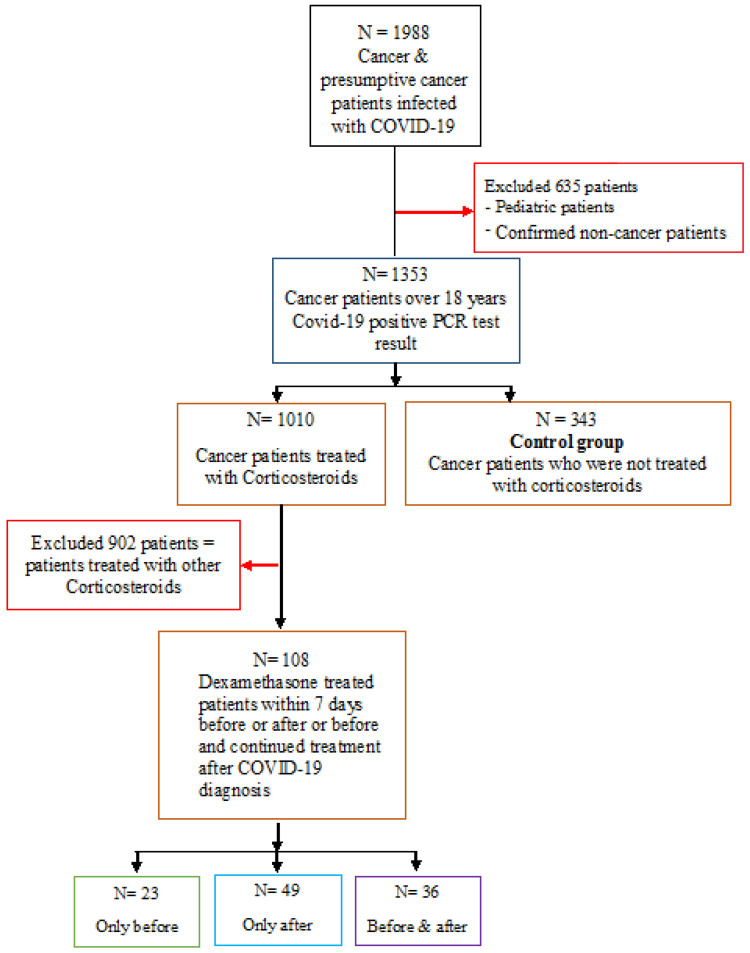
Summary of sampling strategy, inclusion and exclusion criteria.

We conducted a retrospective analysis on adult cancer patients between the ages of 18 and 91 years at King Hussein Cancer Center (KHCC). All patients were Middle Eastern, comprising both Jordanians and non-Jordanians seeking treatment at KHCC and conforming to KHCC’s treatment protocols. All available records at the KHCC Cancer Registry were examined. From this dataset, we identified cancer patients with a verified COVID-19 PCR test result (N = 1988). Only 1353 adult cancer patients with a positive COVID-19 PCR test remained after eliminating children, non-cancer patients, and duplicate records. From the 1010 cancer patients receiving corticosteroid therapy, we included all the patients who received dexamethasone within 7 days before and/or after confirmed positive COVID-19 PCR results (N = 108), and the control sample included all the patients who did not receive any corticosteroid treatment before or after a COVID-19 diagnosis (N = 343). The 7-day window of dexamethasone treatment was applied in order to include only the patients for whom dexamethasone was a relevant factor during their COVID-19 infection [20]. Figure 1 shows the criteria of exclusion resulting in the final sample size. The 108 dexamethasone-treated patients were composed of 23 patients who received dexamethasone within 7 days before their COVID-19 diagnosis, 49 patients receiving dexamethasone within 7 days after diagnosis, and 36 patients receiving dexamethasone within 7 days before and after diagnosis.

The types of cancers were divided into two major categories: solid tumors and hematological malignancies. The solid tumor group consisted of cancers in almost all systems and were distributed mainly according to the most prevalent types (lung, breast, and colorectal). Hematological tumors included different types of leukemia, lymphoma, and multiple myeloma. The dexamethasone dosage varied for the aforementioned cancers depending on the patient’s health, cancer type, the chemotherapy administered, and whether it was used as a treatment or adjuvant therapy. Patients in this study were given doses of dexamethasone within the 4–40 mg range, which is within the therapeutic range used at KHCC.

### 2.2. Groups of Patients According to Their Dexamethasone Healthcare Treatment Plan and COVID-19 Infection Onset

Based on the timing of dexamethasone administration relative to the COVID-19 diagnosis, the dexamethasone treatment group included three subgroups. The first subgroup was cancer patients who were on active dexamethasone treatment within 7 days before their positive COVID-19 PCR test result, but “stopped” receiving the medication after a COVID-19 diagnosis. This subgroup was given the abbreviation (DxB) which stands for “dexamethasone treatment before”.

The second subgroup was cancer patients who started dexamethasone treatment within 7 days after their positive COVID-19 PCR test result. This subgroup was given the abbreviation (DxA) which stands for “dexamethasone treatment after”. Finally, the third subgroup was patients who were already on dexamethasone treatment within 7 days before their positive COVID-19 PCR test result and continued the treatment for at least 7 more days after the positive test result. This subgroup was called (DxBA) which stands for “dexamethasone treatment before and after”.

### 2.3. COVID-19 Disease Severity Indicators

The main indicators of the severity of COVID-19 infections studied were the need for ventilation assistance and/or mortality within 28 days of a COVID-19 diagnosis. Proportions of patients admitted to hospital and to the ICU were also compared between treatment and control groups. Yet, since 96 out of the 104 patients admitted to hospital in the whole sample were admitted on the same day of their COVID-19 diagnosis, probably for monitoring, we cannot consider this as an indicator of severity. Admission to an ICU might be a better indicator of severity.

Ventilation assistance and mortality within 28 days were combined into a composite outcome in a multivariable Cox proportional hazards model, whereas ICU or hospital admissions were used as predictive variables in this model to account for admission causes other than a COVID-19 infection. We could not use admission to the ICU as an outcome variable since admission dates were not available. In addition, the final model had a higher accordance between predicted and actual outcomes, and had a higher likelihood ratio test when both admissions to a hospital and an ICU were included in the model (as predictive variables). Other confounding predictive variables in this model included age, gender, cancer type, and the presence of comorbidity.

### 2.4. Effects of Dexamethasone Treatment on Laboratory Parameters in COVID-19-Infected Cancer Patients

To investigate the effects of a dexamethasone healthcare treatment plan on the severity of a COVID-19 infection using blood test indicators, we chose the most relevant blood tests that were affected by a COVID-19 infection. These tests were the C-reactive protein (CRP) (normal range ≤ 8 mg/L), ferritin (normal range female 4.63–204.0 ng/mL, male 21.81–274.6 ng/mL), D-dimer (normal range < 0.5 µg/mL-FEU), and fibrinogen (normal range 200–400 mg/dL) [21].

### 2.5. Statistical Analysis

Patient status and the date of the last visit were obtained from medical records. This research surveyed 451 adults with SARS-CoV-2 from September 2020 to January 2022. Survival periods were calculated from the date of the COVID-19 diagnosis to the date of either last follow-up or mortality, and patients who were alive or lost to follow-up were censored. All data manipulation and statistical tests were performed using R 4.1.2 (2021) software [22].

Age was summarized with the median and interquartile range. Categorical variables were summarized with frequencies and percentages of the total in each group. For continuous variables, the Mann–Whitney U (M–W) test was used, and for categorical variables, either Fisher’s exact test (F.E) or the Pearson’s chi-squared test was used. The choice between these two tests was based on the expected values, where Fisher’s exact test was used when the expected value in at least one cell in the contingency table was <5. Each treatment group was compared to the control and to other treatment groups, and all the reported *p*-values for these analyses were adjusted for multiple testing with the Benjamini–Hochberg method, which controls the false discovery rate (FDR). A *p*-value < 0.05 was considered significant.

Kaplan–Meier curves were fitted with the “survfit” function in the “survival” package and plotted with “ggsurvplot” in the “survminer” package in R. The multivariable Cox proportional hazards model was fitted using the “coxph” function in the “survival” package in R. The event (outcome) in this analysis was considered to be either death or requiring ventilation assistance within 28 days of the COVID-19 diagnosis. The predictive variables were the dexamethasone treatment group, in addition to chronic conditions, the type of cancer, and gender. Admissions to the ICU and the hospital were also entered into the model as predictive variables, as explained in the above discussion of COVID-19 severity indicators. In order to test the effect of a dexamethasone dose on survival, separate Cox proportional hazards models were fitted for each dexamethasone treatment group. The analysis was performed in R software using the function “coxph” in package “survival”.

The blood test results were taken within 28 days after the positive COVID-19 test date and the data were tested for normality. Since some of the groups were not normally distributed for each blood parameter, the differences between the groups were tested with the Kruskal–Wallis test followed by a pairwise Wilcoxon rank sum test. The distributions of these variables were illustrated with boxplots.

## 3. Results

### 3.1. Effect of Dexamethasone Treatment on COVID-19 Disease Prognosis

This study included 451 adult cancer patients who were diagnosed with SARS-CoV-2 in the period between September 2020 and January 2022. Out of the 451 patients, 108 were in the study group and 343 in the control group. The mean age of the entire sample was 57.2 years (SD 16.4), and the median was 58 years with a range 19–92 years. There was a slightly greater percentage of female patients (242/451, 53.7%) than male patients, but neither age nor gender showed any significant difference between control and treatment groups. Most patients had solid tumors (408/451, 90.7%) which is the case in both the control and study group. Demographics are listed in Table 1.

Table 2 shows that significantly more patients in the dexamethasone group as a whole required ventilation assistance (14/108 (13%), *p*-value < 0.05) when compared to the control group (7/343 (2%)). Admissions to the hospital and the ICU were also significantly higher in the dexamethasone-treated group (hospital: 68/108 (63%), *p*-value < 0.05), (ICU: 14/108 (13%), *p*-value < 0.05) compared to the control group (hospital: 59/343 (17%)), (ICU: 2/343 (1%)), respectively. Similarly, the mortality rate was significantly higher in the dexamethasone group (39/108 (36%), *p*-value < 0.05) compared to the control group (36/343 (10%)).

Out of the 108 treated patients, 23 patients were on active medication within 7 days before their COVID-19 diagnosis (DxB), 49 patients started dexamethasone therapy within 7 days after their COVID-19 diagnosis (DxA), and 36 patients were on active medication within 7 days before their COVID-19 diagnosis and continued on dexamethasone treatment after the diagnosis (DxBA). The percentage of patients who needed ventilation assistance, those who were admitted to the hospital or to the ICU, and those who died within 28 days of their COVID-19 diagnosis were significantly higher in the subgroup (DxB) compared to the control group, *p*-value < 0.05 (Table 3).

Similarly, our data show a significant increase in the admission to the hospital or the ICU and a mortality rate within 28 days of COVID-19 infection in the patients who received dexamethasone treatment only after being diagnosed with COVID-19 (DxA) compared to the control group (*p*-value < 0.05). Nevertheless, there was no significant difference in needing ventilation assistance between these two groups (Table 3). As for the subgroup of patients who were on dexamethasone treatment before their COVID-19 infection and who continued their treatment after the diagnosis (DxBA), there was a significant increase in requiring ventilation assistance and admissions to the hospital and the ICU compared to the control group (*p*-value < 0.05). There was no significant difference in the mortality rates within 28 days from COVID-19 infection between these two groups (Table 3).

### 3.2. Comparison within the Three Subgroups of Dexamethasone

The three subgroups were compared to each other relative to their COVID-19 severity indicator variables. It was found that cancer patients in the (DxB) subgroup had a significantly worse outcome than the (DxA) subgroup, both in terms of the need for ventilation assistance (26% vs. 4%, *p*-value < 0.05) and mortality within 28 days of a COVID-19 diagnosis (74% vs. 31%, *p*-value < 0.05). In addition, the (DxB) subgroup exhibited a significantly higher death rate when compared to the (DxBA) subgroup (74% vs. 19%, *p* < 0.05). Our data also showed that there was no significant difference between the subgroups (DxA) and (DxBA) when compared to each other (*p*-value > 0.05) (Table 4).

### 3.3. Outcome and Survival of Various Subgroups

**Figure 2:**
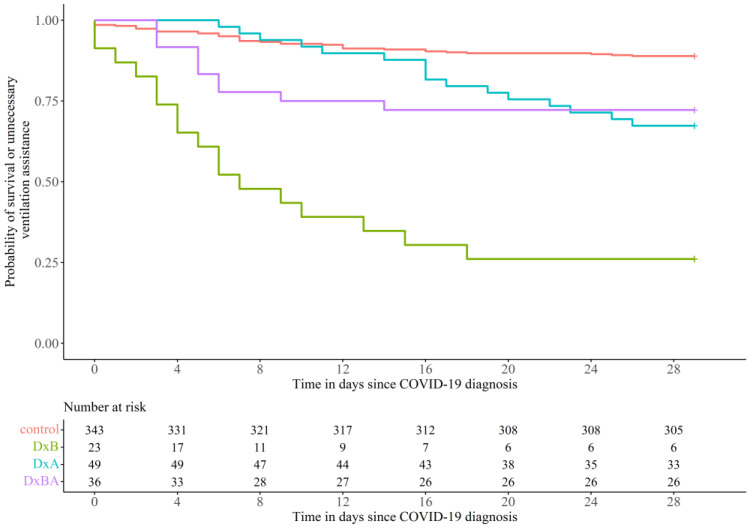
Kaplan–Meier curve for dexamethasone-treated patient subgroups compared to the control group. Control (Red, N = 343, events = 38), patients receiving Dexamethasone only before (DxB) COVID-19 diagnosis (Green, N = 23, events = 17), patients receiving Dexamethasone only after (DxA) COVID-19 diagnosis (Blue, N = 49, events = 16), and patients receiving Dexamethasone before and after (DxBA) COVID-19 diagnosis (Purple, N = 36, events = 10).

Mortality within 28 days after a COVID-19 diagnosis or the need for ventilation assistance in the subgroup (DxB) was found to be the highest at 73.9% (17/23). This was followed by the subgroup (DxA) at 32.7% (16/49), whereas the subgroup (DxBA) demonstrated the lowest mortality and need for ventilation among the three subgroups (27.8% (10/36)) (Figure 2). All three subgroups were relatively worse than the control group (11.1% (38/343)). The effect of treatment regimen on survival was tested with a multivariable Cox proportional hazards model.

### 3.4. Multivariable Cox Proportional Hazards Model

Age, admission to the ICU or hospital, and receiving dexamethasone only before a COVID-19 diagnosis were found to be significantly associated with risk of mortality or ventilation assistance within 28 days of a COVID-19 diagnosis. Receiving dexamethasone only before diagnosis was significantly associated with a 5.8-fold higher risk of experiencing the outcome within 28 days compared to the control group (*p* value = 2.84 × 10^−6^). Each year, an increase in the age of patients was significantly associated with a 3% increased risk of mortality or requiring ventilation (hazard ratio 1.03, 95% CI 1.01–1.05, *p* value = 0.00059). Admission to the ICU was significantly associated with a 240% increased risk of mortality or requiring ventilation (hazard ratio 3.4, 95% CI 1.6–7.4, *p* value = 0.00215). Admission to the hospital was also significantly associated with a 630% increased risk of mortality or the requirement of ventilation (hazard ratio 7.3, 95% CI 4.2–13.0, *p* value = 6.18 × 10^−12^) (Table 5). The presence of comorbidities, such as diabetes, heart failure, history of stroke, myocardial infarction, obstructive lung disease, acute kidney injury, and hypertension, was not found to be significantly associated with risk of mortality within 28 days after a COVID-19 diagnosis or the requirement of ventilation assistance.

### 3.5. Effect of Dexamethasone Treatment Dose

Next, we investigated the effect of a dose of dexamethasone before and/or after a COVID-19 diagnosis on patient survival. Separate Cox proportional hazards models were fitted for each dexamethasone treatment group.

There was no significant effect before or after the dose was administered, or any interaction between the two doses, at the time of the event in any of the treatment groups. For the (DxB) group, the hazard ratio for dexamethasone dose was 0.864 (0.720–1.036), z = −1.575, and *p* = 0.115. For the (DxA) group, the dose hazard ratio was 0.912 (0.756–1.100), z = −0.96, *p* = 0.337. Finally, the hazard ratios for the (DxBA) group for the dose before, after, and the interaction between the doses before and after were as follows: before: 1.079 (0.673–1.727), z = 0.314, *p* = 0.753; after: 1.094 (0.551–2.173), z = 0.257, *p* = 0.797; interaction before and after: 0.993 (0.500–1.973), z = −0.256, *p* = 0.798.

### 3.6. Effect of Dexamethasone Healthcare Treatment Plan on Laboratory Test Results

**Figure 3:**
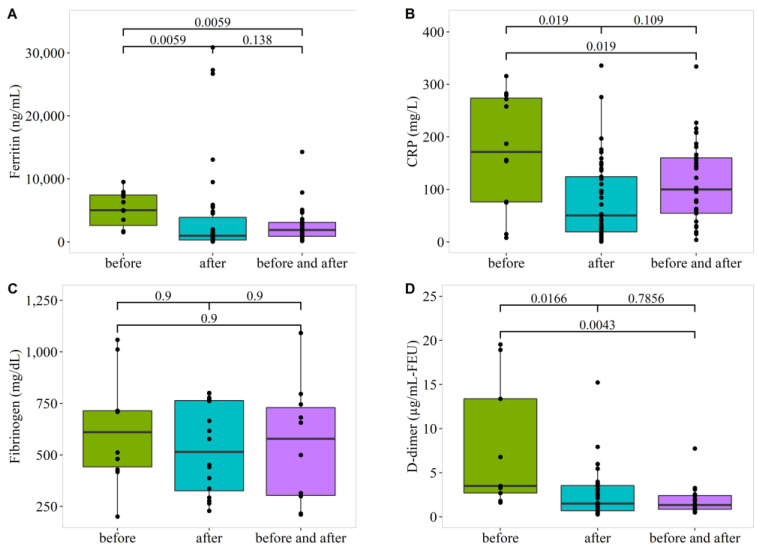
Effect of dexamethasone healthcare treatment plans on laboratory test results. Boxplots of laboratory test results for Dexamethasone healthcare treatment plans. (**A**) Ferritin (ng/mL), (**B**) CRP (mg/L), (**C**) Fibrinogen (mg/dl), and (**D**) D-dimer (µg/ml-FEU). Patients treated with Dexamethasone before COVID-19 infection (DxB). Patients treated with Dexamethasone after COVID-19 infection (DxA). Patients treated with Dexamethasone before COVID-19 infection and continued their treatment after diagnosis (DxBA). Kruskal-Wallis test was used to test the differences in the distributions in laboratory readings between the different Dexamethasone treatment regimens, and pairwise comparisons were corrected for multiple testing with Benjamini–Hochberg method.

Patients treated with dexamethasone before COVID-19 infection (DxB) had significantly higher median blood levels of CRP, ferritin, and D-Dimer compared to patients in the other two groups (DxA and DxBA). The median CRP level was 171.5 mg/L vs. 50.4 mg/L and 100 mg/L; ferritin: 4960 ng/mL vs. 985.5 ng/mL and 1886 ng/mL; and D-dimer: 3.5 µg/mL-FEU vs. 1.5 µg/mL-FEU and 1.34 µg/mL-FEU for the (DxB), (DxA), and (DxBA), respectively. Furthermore, our data showed no significant differences between the two subgroups (DxA) and (DxBA) in the results of CRP, ferritin, and D-Dimer (Figure 3A,B,D). Subgroups (DxA) and (DxBA) in the results of CRP, ferritin, and D-Dimer (Figure 3A,B,D). The median fibrinogen levels (610.5 mg/dL vs. 452 mg/dL and 578.5) were not significantly different among the three subgroups (Figure 3C).

## 4. Discussion

In this study, we investigated the impact of dexamethasone pretreatment on COVID-19 prognosis in cancer patients. Specifically, we studied whether dexamethasone treatment before or after a COVID-19 diagnosis, or both, was linked to better or worse outcomes in terms of mortality within 28 days after the COVID-19 diagnosis and the need for ventilation assistance, in addition to admission to the hospital and the ICU. The three dexamethasone treatment subgroups were compared with a COVID-19-infected cancer patient control group that was not receiving corticosteroids.

Our data showed that all the indicator variables studied (need for ventilation assistance, admission to hospital and/or ICU, and mortality rates within 28 days of COVID-19) were significantly better in the control group compared to the dexamethasone-treated cancer patients. This is not an unlikely finding given that patients in the control group did not receive any corticosteroid medication before or after their COVID-19 diagnosis, which may be an indirect indicator that they were in a healthier state than those in the dexamethasone-treated group. To confirm this hypothesis, we compared the Eastern Cooperative Oncology Group (ECOG) performance status for the control patients’ group with the dexamethasone-pretreated cancer patients’ group. The ECOG performance status ranks the functioning and ability to care for the patient on a scale of zero to five, where the best score equals zero. The comparison revealed that the median ECOG scores of the control group were considerably higher than those of the three subgroups, indicating that the health status of patients in the control group was better compared to that of the dexamethasone-pretreated patients’ group. Based on this observation, we decided to evaluate the dexamethasone-treated subgroups against one another to rule out the possibility that the control group of cancer patients was in better health, to begin with. We also compared each subgroup to the control group to determine whether the subgrouping of patients contributed to the statistically significant difference between the control group and each subgroup.

The results showed that regardless of the dexamethasone treatment plan, all subgroups who received dexamethasone (DxB, DxA, and DxBA) had significantly higher rates of admission to the hospital or ICU compared to the control group, *p*-value < 0.05. This shows that dexamethasone treatment in COVID-19-infected cancer patients may be associated with poor performance outcomes in the aforementioned indicators, which was similar to other recently published studies monitoring non-cancer patients [20,23]. Furthermore, the need for ventilation assistance was significantly increased among (DxB) patients and (DxBA) patients compared to the control group (*p*-value < 0.05). However, ventilation assistance was not significantly increased in the subgroup of patients who started dexamethasone only after their diagnosis (DxA) compared to the control group (*p*-value = 0.520). This finding supports several publications recommending the use of dexamethasone after COVID-19 infection to minimize the need for mechanical ventilation [24,25,26]. As for the effect of dexamethasone treatment on inflammation-related lab results, we found that (DxB) patients had the highest levels of CRP, ferritin, and D-Dimer levels compared to (DxA) and (DxBA), as was previously reported in COVID-19-infected cancer patients [27].

More importantly, our intra-statistical analysis of the dexamethasone-treated subgroups revealed that the (DxB) regimen was substantially related to an increased risk of mortality and requiring ventilation assistance compared to the (DxBA) and (DxA) regimens, and that this association was independent of the dexamethasone dose. Consequently, our data strongly suggest that discontinuing dexamethasone medication had a negative impact on the overall survival and patient’s well-being.

The small sample size is one of the limitations of this study; consequently, additional research is required to validate these results. In addition, a comprehensive prospective comparison between early and late administration of dexamethasone is necessary to determine the optimal administration timing of dexamethasone.

## 5. Conclusions

In conclusion, our study assessed the impact of a dexamethasone medication treatment plan on the severity of COVID-19 in cancer patients before or after COVID-19 infection, or both. Based on our data, it is evident that continuing dexamethasone treatment upon a COVID-19 diagnosis may increase survival rates and improve the prognosis for COVID-19, as opposed to terminating the medication after a COVID-19 infection in dexamethasone-pretreated cancer patients.

## Figures and Tables

**Table 1:** Demographics of the study population showing no significant differences in the characteristics of both groups.

Groups Demographic	Control N (%)	Dexamethasone N (%)	*p*-Value
Total (N = 451)	343 (60.5%)	108 (19.1%)	
**Age**			
Median (years)	58.0	58.5	0.438
**Gender**			
Male	151 (44%)	58 (54%)	0.0992
Female	192 (56%)	50 (46%)	
**Cancer type**			
Solid	318 (93%)	91 (87%)	0.0846
Hematological	25 (7%)	14 (13%)	

**Table 2:** Indicator variables of the study population.

Indicator Variables	Control	Dexamethasone	*p*-Value
	N (%)	N (%)	
Total (*n* = 451)	343 (60.5%)	108 (19.1%)	
**Ventilation assistance**			<0.05
Yes	7 (2%)	14 (13%)	
No	336 (98%)	94 (87%)	
**Admitted to hospital**			
Yes	59 (17%)	68 (63%)	<0.05
No	284 (83%)	40 (37%)	
**Admitted to ICU**			
Yes	2 (1%)	14 (13%)	<0.05
No	341 (99%)	94 (87%)	
**Mortality within 28 days of COVID-19**			<0.05
Yes	36 (10%)	39 (36%)	
No	307 (90%)	69 (64%)	

**Table 3:** Effect of dexamethasone treatment before COVID-19 infection on disease indicators.

Groups	Control	Dexamethasone Treatment before COVID-19 Infection or within 7 Days from Testing	
**Indicator variables**	N (%)	N (%)	*p*-value
Total (*n* = 451)	343 (76.1%)	23 (5.1%)	
**Ventilation assistance**	7 (2%)	6 (26%)	<0.05
Yes			
No	336 (98%)	17 (74%)	
**Admitted to hospital**			
Yes	59 (17%)	11 (48%)	<0.05
No	284 (83%)	12 (52%)	
**Admitted to ICU**			
Yes	2 (1%)	6 (26%)	<0.05
No	341 (99%)	17 (74%)	
**Mortality within 28 days of COVID-19**			
Yes	36 (10%)	17 (74%)	<0.05
No	307 (90%)	6 (26%)	

*p*-value: from Fisher’s exact test or Pearson’s chi-squared test where appropriate. All *p*-values in the table are adjusted for multiple testing using the Benjamini–Hochberg method.

**Table 4:** Comparison between different dexamethasone treatment subgroups.

Groups	Dexamethasone Treatment before vs. after COVID-19 Infection		Dexamethasone Treatment before vs. before and after COVID-19 Infection		Dexamethasone Treatment after vs. before and after COVID-19 Infection	
Indicators variables	DxB vs. DxA	*p*-value	DxB vs. DxBA	*p*-value	DxA vs. DxBA	*p*-value
Ventilation assistance	26% vs. 4%	<0.05	26% vs. 17%	0.586	4% vs. 17%	0.112
Admitted to hospital	48% vs. 67%	0.185	48% vs. 67%	0.244	67% vs. 67%	1
Admitted to ICU	26% vs. 10%	0.163	26% vs. 8%	0.139	10% vs. 8%	1
Mortality within 28 days of COVID-19	74% vs. 31%	<0.05	74% vs. 19%	<0.05	31% vs. 19%	0.362

**Table 5:** Hazard ratios from multivariable Cox proportional hazards regression.

Variable	Hazard Ratio (95% CI)	z	*p*-Value
age	1.03 (1.01–1.05)	3.435	<0.005
diabetes	1.8 (0.99–3.1)	1.943	0.0521
heart failure	1.2 (0.37–4.0)	0.335	0.738
history of stroke	NA	NA	NA
myocardial infarction	0.4 (0.04–3.6)	−0.814	0.416
Obstructive lung disease	2.0 (0.42–9.14)	0.852	0.394
acute kidney injury	NA	NA	NA
Hypertension	0.6 (0.3–1.1)	−1.61	0.107
Gender	0.8 (0.5–1.3)	−0.745	0.456
admission to ICU	3.4 (1.6–7.4)	3.069	<0.005
admission to hospital	7.3 (4.2–13.0)	6.875	<0.005
Type of cancer (hematological vs. solid)	1.8 (0.9–3.6)	1.592	0.111
Dexamethasone before vs. control	5.8 (2.8–12.0)	4.682	<0.005
Dexamethasone after vs. control	0.9 (0.5–1.7)	−0.411	0.681
Dexamethasone before and after vs. control	0.8 (0.3–1.7)	−0.669	0.504

The outcome variable is either death or requiring ventilation assistance within 28 days of COVID-19 diagnosis. The sample of 451 patients was included (343 control and 108 receiving dexamethasone). NA: Very few or no individuals apply.

## Data Availability

The data presented in this study are available by request from the corresponding author. The data are not publicly available for ethical reasons.

## Abbreviations

SARS-CoV-2 (severe acute respiratory syndrome coronavirus 2), ICU (intensive care unit), PCR (polymerase chain reaction), FEU (fibrinogen-equivalent units)
